# Change in Sexual and Reproductive Health Knowledge among Young Women Using the Conversational Agent “Nthabi” in Lesotho: A Clinical Trial

**DOI:** 10.21203/rs.3.rs-3788533/v1

**Published:** 2023-12-28

**Authors:** Elizabeth Nkabane-Nkholongo, Mathildah Mokgatle, Timothy Bickmore, Clevanne Julce, David Thompson, Brian JAck

**Affiliations:** Sefako Makgatho University of Health Sciences; Sefako Makgatho University of Health Sciences; Northeastern University; University of Massachusetts Chan Medical School; University of Oklahoma Health Sciences Center; Boston University/Boston Medical Center

**Keywords:** conversational agent technology, women’s health education, mHealth adaptation, health information technology, health education in Africa

## Abstract

**Background.:**

Young women worldwide face problems like unwanted pregnancy and sexually transmitted infections. Providing sexual and reproductive health education to this population remains a priority. It is unknown if using digital health interventions to deliver health education in human resource-constrained settings is effective.

**Methods.:**

We conducted a clinical trial of the Nthabi intervention to determine participant’s knowledge before and after discussion of family planning, folic acid and healthy eating among young women aged 18–28 years in two rural districts of Lesotho who used the Nthabi conversational agent system on either smartphones or tablets for up to six weeks. The number of correct pre- and post-test responses were compared using generalized linear models that directly estimated the proportions and percentages of correct responses.

**Results.:**

Of the 172 participants enrolled, the mean age was 22.5 years, 91% were unmarried, 69% completed high school, 23% were unemployed and 66% were students. The mean number of interactions with Nthabi was Family planning was chosen to be discussed by 82 (52.2%), of the 172 participants and of those, 49 (59.8%) completed the content on this topic, and 26 (53.1%) completed the post-test. For the 11 questions about family planning, there were 717 (76.6%) correct responses on the pre-test and 320 (89.9%) on the post-test (p = 0.0233). Folic acid was chosen to be discussed by 74 (47.1%) of 172 participants, and of those, 27 (36.5%) completed the content on this topic, and all 27 (100%) completed the post-test. For the 5 questions about folic acid use, there were 181 (45.3%) correct responses on the pre-test and 111 (71.6%) on the post-test (p < 0.0001). The number of correct responses on the post-test was positively associated with the number of sessions that the participant engaged with Nthabi.

**Conclusion.:**

The Nthabi conversational agent system increased knowledge of family planning methods and folic acid use among young women in Lesotho. Digital health interventions like Nthabi offer new opportunities to deliver reproductive health information in countries that have limited human resources for health.

**Trial Registration:**

ClinicalTrials.gov ID: NCT04354168

## BACKGROUND

Sexual and reproductive health (SRH) remains a global public health challenge among the 1.8 billion adolescents and young people ages 15–24 worldwide. They face problems like unwanted pregnancy, and sexually transmitted infections (STIs) including human immunodeficiency virus (HIV) infections. This population accounts for 42% of new HIV infections globally, and 4 of 5 young people with HIV live in sub-Saharan Africa.

Lesotho has the second-highest HIV prevalence in the world, at 22.7%, and one of the highest HIV incidences among adolescent girls and young women at 0.33% annually. The maternal mortality rate in Lesotho of 544/100,000 live births is the second highest in Southern African Development Community countries. In Lesotho, this age group commonly reports low SRH knowledge and engages in risky sexual behaviours such as early sexual debut (14 years), unprotected sex, and multiple sexual partners resulting in susceptibility to STIs, including HIV, unintended pregnancy, and unsafe abortions. Young people in low and middle-income countries (LMIC) have limited access to SRH information and face myriad challenges accessing SRH services such as providers’ judgemental approach and fear of stigmatization. In Lesotho, adolescent girls and young women commonly report low sexual and reproductive health knowledge. On a survey about comprehensive knowledge on HIV, only 30.7% of young women displayed comprehensive knowledge.

The rapid diffusion of mobile technologies among young people in Lesotho^,^ including smartphones, social media or text-messaging has the potential to deliver safe and effective SRH health education and could serve to expand the reach of health professionals in human resource-restricted settings such as Lesotho. These technologies could be used to support large-scale health promotion efforts that could be cost-effective.^,^

Embodied Conversational Agents (ECAs) are computer-based animated characters designed to simulate face-to-face human interactions. They have the potential to automate tasks, improve access to healthcare services and reduce health professionals’ workload.^,^ In the United States, an ECA named “Gabby,” designed to deliver SRH to reproductive age African American women in the United States, demonstrated significant reduction in reproductive health risks in a randomized clinical trial.^,^ Subsequently, our research team adapted Gabby for use in Lesotho (named the “Nthabi Preconception Health Promotion Application” or “Nthabi”). The Nthabi character is a Mosotho nurse midwife designed to deliver SRH education to young women in Lesotho.

We previously reported survey results of the young women who used Nthabi showing that they perceived Nthabi to be effective, efficient, and culturally appropriate for delivering sensitive SRH information. Respondents agreed that Nthabi helped them make decisions and could improve the delivery of health education. They reported it was easy to use and well organized. Most respondents reported that they intend to use it beyond the study period and would encourage others to use the App.

This paper reports changes in participant’s knowledge, which was assessed before and after using Nthabi among the 172 young women enrolled in the study.

## METHODS

### Study Aim, Study Design, Population and Setting.

The aim of the study was to determine if the Nthabi Health Promotion Application improves participant’s knowledge after using Nthabi compared with before using the App. We enrolled 172 young women from the two rural districts of Leribe and Berea in the mountainous, low-middle income country of Lesotho in southern Africa.

### Recruitment.

The research team recruited participants by posted messages on social media (e.g., WhatsApp and Facebook) that described the study and asked potential participants to contact the research team to discuss enrolling in the study., A local non-governmental organization called *Help Lesotho* that offers mentorship programs to adolescent girls and young women in the Leribe district, reached out to the research team after seeing the social media posting and offered to disseminate the recruitment announcement to their clients.

Second, the research team approached young women while they were waiting for consultation in the Adolescent Health Corners [e.g., clinics], the HIV, and Mother and Child Health ambulatory clinical departments at the Berea and Leribe government district hospitals. Third, students were approached at the Leribe Vocational School and the Limkokwing University of Technology to identify those interested in participating.

### Eligibility and Enrollment.

The inclusion criteria were: (1) Basotho women aged 18–28 years who were from the districts of Leribe or Berea and who accessed health services in these two districts, (2) self-reported ability to read and understand spoken English, (3) have access to an Android smartphone, (4) able to access internet and Wi-Fi at least once at the end of the study.

### Informed Consent, Enrollment.

Once eligibility criteria were established, the research team explained the purpose of the study, potential risks and benefits, reimbursement for travel costs, and the right to withdraw from the study at any time. After addressing any questions, participants were asked to sign an informed consent.

The research team then assisted the participants to download the App on their mobile phones. Participants who were unable to download the App were loaned a Lenovo© Android 11 OS platform tablet to use for six weeks. Participants created a unique username and password and were shown how to log on to their mobile phone or tablet. Participants then began interactions with Nthabi and were encouraged to use the app at least once daily at their convenience for six weeks.

### Baseline Data Collection.

Socio-demographic information was collected (age, marital status, educational level, employment status, recruitment site, and district).

### Nthabi Intervention Description.

We previously described [24] how we adapted an ECA used in the United States (“Gabby”) to create an agent (“Nthabi”) whose physical characteristics and language, and the clinical contents it presents, are appropriate for Lesotho. ^24^ Adaptations explored physical and cultural alternatives including the character’s sex, age, occupation, name, physical appearance (hairstyle, clothing), language and speech patterns, and personality believed to resonate with young Basotho women.

**Figure 1:**
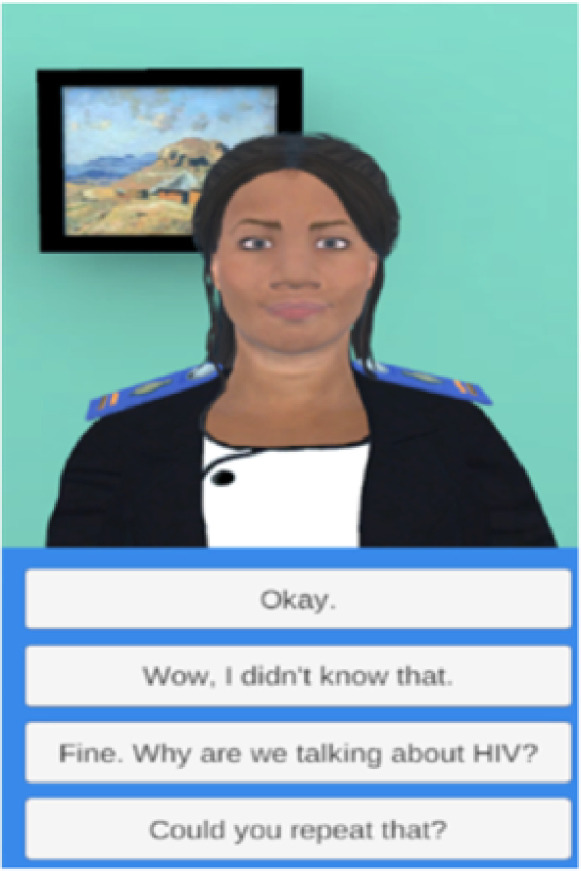
Nthabi Health Education App Participant Interface

The final Nthabi’s appearance and persona represent a Mosotho professional nurse midwife wearing a Lesotho nurse’s uniform. Her hairstyle (braids), complexion (medium, similar to the local population), facial expressions (calm and gentle), and mannerisms (a humble professional with a sense of humour) are relatable to young women in Lesotho (Fig. 1).

Nthabi’s language is English, as the English literacy rate among young women in Lesotho is above 90%, and English is spoken in health settings.

To establish the clinical topics to be included in the system, Lesotho Ministry of Health key informants recommended five sexual reproductive health topics for young women (family planning, HIV, tuberculosis (TB), healthy eating and use of folic acid). The research team then used the Lesotho National Clinical Guidelines to create evidence-based dialogue for use in each of the five content areas discussed in Nthabi interactions.^16^

Participants chose from a list of the five health topics offered by Nthabi. Using conversational dialogue, Nthabi describes why the topic is important and offers suggestions about how to take action on it. Participants engage by selecting a response from a multiple-choice menu updated at each turn of dialogue. For family planning, folic acid and healthy eating, Nthabi requests that participants take a knowledge pre-test before providing health education, and when they completed the topic, the participants were asked to take a post-test. There were no pre-post interaction questions for HIV and TB as these topics were designed to test the impact of motivational dialogue on health behaviors. These data will be presented separately.

Finally, technical adaptations were required to deploy Nthabi on smartphone screens rather than computer screens as done in the Gabby prototype. We constructed the app to be fully downloadable to users’ mobile phones so that its availability would not require Wi-Fi access. Usage and information about the health content discussed would be downloaded to the server when the user is in a Wi-Fi environment. Nthabi was made available on Google Play store for download on mobile phones or tablets.

### Data Collection.

When the participant is in a Wi-Fi environment, the data collected through conversations with Nthabi is automatically downloaded to a secure server collecting the following metrics: number of logins, number of interactions with the system and the number of topics completed and pre and post-test results. Participants were invited to return to the recruitment site to access the internet at the completion of the study to facilitate downloading interaction data to the server, and to return the loaned tablets.

### Data Storage and Analysis.

Data from the server were extracted, captured on an Excel spreadsheet, and stored on a password-protected computer. Data were analysed using SAS statistical software (v9.4). The number of correct pre- and post-test responses were compared using generalized linear models that directly estimated the proportions (and percentages) of correct responses. For individual items, the models accounted for the pre-and post-test responses being paired for each participant. Models that compared paired responses across all multiple items also accounted for the clustering of multiple responses within individual participants. P-values of < 0.05 were considered statistically significant.

### Participant Incentives.

All participants received 50 Maloti (approximately $5) to cover the cost of using phone data. Participants using tablets received an additional 50 Maloti to cover their travel back to the recruitment sites to return the devices.

### Ethical Clearance.

The study was conducted according to the Consolidated Standards of Reporting Trials (CONSORT). Ethical clearance was obtained from the Sefako Makgatho University of Health Sciences Ethics Review Committee (SMUREC/H/343/2021: PG), Boston University Research Institutional Review Board (IRB Number: H-40268), the Lesotho Ministry of Health Research Ethics Committee (ID 145–2021), and permission was obtained from the study recruitment sites.

## RESULTS

**Figure 2:**
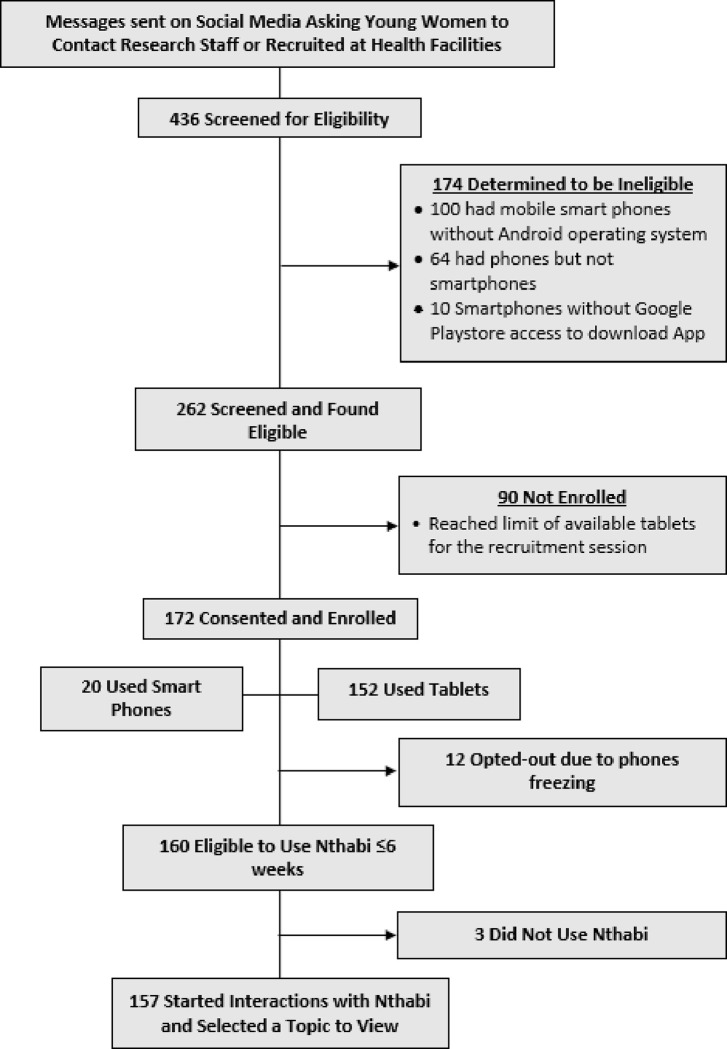
Consort Diagram

As shown in the CONSORT diagram (Fig. 2), the research team screened 436 young women for eligibility.^19^ Young women were recruited through social media (e.g., WhatsApp, Facebook) or direct contact at Limkokwing University of Technology (n = 150), Leribe Vocational School (n = 88), Leribe Health facilities (n = 55), Berea Health Facilities (n = 84) and *Help Lesotho* (n = 59).

Of those screened, 174 were ineligible due to having smartphones without an Android operating system, 64 had phones that were not smartphones and 10 had Huawei Android Smartphones without access to the Google Play store. Of those found eligible, 90 others were not enrolled due to reaching the limit on available tablets in that recruitment session. Consequently, 172 participants consented and were enrolled. Of those enrolled, only 20 had sufficient memory on their phones to download the Nthabi App. The 152 unable to download the App received a Tablet device to use.

In the weeks after enrollment, 12 participants opted out of the study because their phones froze or jammed when loading. Therefore, 160 young women used Nthabi, with eight using their phones and 152 using tablets. Of the 160, 157 started interactions with Nthabi and selected a topic to view (Fig. 2).

Table 1 shows the characteristics of the 172 participants who were consented and enrolled. The mean age was 22.5 years (standard deviation of 2.63), 91% were unmarried, 69% had completed high school, 23% were unemployed and 66% were students.

The recruitment sites of participants enrolled were: 34 (20%) from Limkokwing University of Technology, 60 (35%) from Leribe Vocational School, 31 (18%) from Leribe Health Facilities, 7 (4%) from Berea Health Facilities, and 40 (23%) from *Help Lesotho*.

Table 2 shows the number and percent of participants enrolled who selected a health topic to review, completed a topic and those that then took the post-test. Among the three topics, family planning was most frequently chosen to be viewed by participants with 82 (52.2%) selecting this topic, and of those viewing family planning, 49 (59.8%) completed the topic, and 26 (53.1%) completing the post-test.

For folic acid, 74 (47.1%) participants viewed the topic, of those, 27 (36.5%) listened to all the content, and all 27 (100%) completed the post-test. For healthy eating, 76 (48.4%) selected this topic to view, of those, only 15 (19.7%) completed the topic, and 13 (86.7) completed the post-test.

Table 3 shows 91(58.0%) of participants completed at least one topic area with 49 completing family planning, 27 folic acid and 15 healthy eating. Thirty-two participants (20.4%) completed one topic (24 family planning, 7 folic acid and 1 healthy eating); 21 (13.4%) of participants completed two topics (10 family planning, 8 folic acid and 3 healthy eating); and 38 (24.2%) of participants completed three topics (15 family planning, 12 folic acid and 11 healthy eating). Overall, 91 (58.0%) of the participants completed at least one topic area.

Table 4 shows the results of the folic acid pre and post-test questions. Overall, of the participants responding to the 5 questions on the pre-test with 181 (45.3%) questions answered correctly on the pre-test and 111 (71.6%) answered correctly on the post-test (p < 0.0001). Participants improved significantly on each question. For example, question 4, “folic acid reduces risks of birth defects” (true of false) showed an increase in correct responses from 64 (80%) to 30 (96.8%), p = 0.0029. For question 5 (“How much folic acid should women of childbearing age take daily to prevent birth defects?”) showed an increase in correct responses from 10 (12.7%) vs 21 (67.7%), p < 0.0001).

Table 5 shows the results of family planning pre and post-test questions. Overall, of the participants responding to the 11 questions on the pre-test with 717 (76.6%) questions answered correctly on the pre-test, and 320 (89.9%) answered correctly on the post-test (p = 0.0233). Participants improved significantly on each question. For example, question 1 “Women can have an operation to avoid having any more children.” (true/false) showed an increase in correct responses from 79 (87.8%) to 32 (97%), p = 0.0461. For question 11, “As an emergency measure, within five days after they have unprotected sexual intercourse, women can take special pills to prevent pregnancy?” increased the percent correct on the pre-test from 69 (83.1%) to 30 (96.8%) on the post-test (p = 0.0106).

Table 6 shows the results pre and post-test questions about the healthy eating content. Overall, of the participants responding to the five questions on the pre-test with 306 (69.5%) questions answered correctly on the pre-test, and 95 (72.6%) answered correctly on the post-test (P = 0.421).

**Figure 3:**
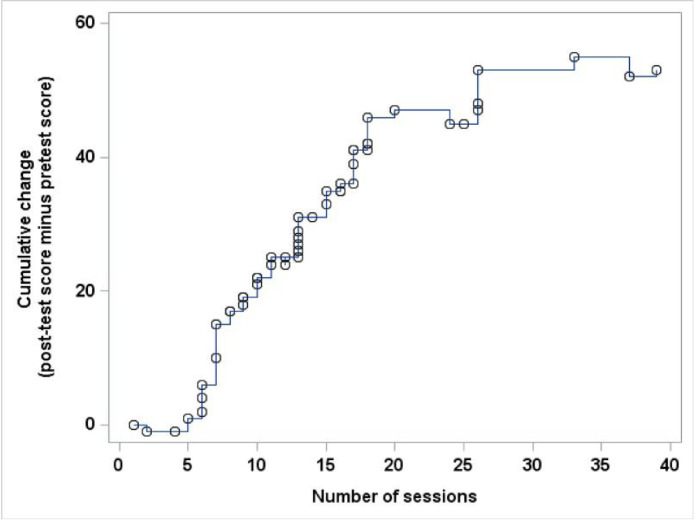
Relationship between the Number of interactions with Nthabi and the Change in the Number of Correct Responses Pre/Post Test The figure shows that the number of correct responses (minus the incorrect responses) increases with increasing number of interactions. The curve flattens because when participants exhausted the content for folic acid, family planning and healthy eating, they were then presented with additional content that did not contain pre- and post questions.

The mean number of interactions (logons) was 8.6 per enrolled participant, with a maximum of 39 interactions. Figure 3 shows the relationship between the number of sessions the participants signed onto Nthabi and the change in the number of correct responses on the pre and post-test. The figure shows that as the number of sessions that the participant engaged with Nthabi increased, the greater the number of correct answers were reported on the post-test.

## DISCUSSION

Young women enrolled in this study demonstrated a significant increase in knowledge about family planning methods and preconception folic acid use after interacting with the Nthabi for up to 6 weeks. These data support the idea that systems like Nthabi have great potential to improve the delivery of sexual and reproductive health education in low and middle-income countries like Lesotho.

The use of m-Health technologies might be particularly important in countries like Lesotho that face severe constraints on human resources for health that limit the delivery of health education and services. Systems like Nthabi could “leapfrog” over currently limited face-to-face health education and could represent the health care of the future in settings such as Lesotho.

Nthabi delivers sexual health information that is non-judgmental and culturally appropriate.^16^ The standardized conversational dialogue might overcome the impact of cultural taboos and social stigma on the use of sexual and reproductive language and conversation, lack of culturally appropriate curricula, and judgmental attitudes of healthcare professionals.^9^

We previously reported that focus group participants who used Nthabi perceived the adaptations to be culturally appropriate, and provided relevant clinical information.^16^ In survey results, clinical trial participants perceived Nthabi to be effective, efficient and helped them make better health decisions. They intend to use and encourage others to use it beyond the study period.^17^

In this report we now show an increase in knowledge of sexual and reproductive health, but our data do not provide evidence that the intervention improves young women’s health-related behaviours or improves health outcomes. Such studies are planned.

Our findings are in concordance with other studies conducted in low-middle-income countries suggesting that health information tools are a feasible method to increase reproductive and sexual health knowledge^2,9,^ and have been shown to be feasible and acceptable for improving health education and knowledge among adolescents and young people. Other studies highlighted the broad potential for digital interventions to enhance health promotion and service delivery towards better sexual health. However this is the first study showing an increase in knowledge resulting from potentially more engaging and effective conversational agent systems in a low and middle income country in southern Africa.

Improved knowledge of health topics improves clinical outcomes in a variety of studies.^9^ Our data support conducting studies to determine if improved knowledge of SRH from interactions with Nthabi results in improved clinical outcomes. For example, future studies can test if improved knowledge of family planning could translate to reduction of unintended pregnancies, which remain unacceptably high among adolescent girls and young women in southern Africa. Unintended pregnancy in young women has major health and social implications and places a substantial economic burden on health care systems.

Nthabi has potential for use as a population health tool at the national or health system level. If Nthabi is available electronically to a population of young women, our data suggest that knowledge of the topics presented will improve as demonstrated using similar conversational agent systems among young African American women in the United States.^15^

There still could be an important clinical impact of increased knowledge of folic acid even if a limited percent of those to whom the technology is made available use it, as shown in this study. For example, assuming increased knowledge of the impact of folic acid use translates into folic acid use before pregnancy, an annual birth rate of 24,881, and a rate of neural tube defect (NTDs) of 28.44 per 10,000 births, would result in 12 fewer babies being born with a NTD in Lesotho each year. Even if only half who complete the content use folic acid, it would still result in 6 few NTDs per year, and the uptake of the folic acid content would likely increase if Nthabi was made available for more than 6 weeks.

Our data also showed that participants did not interact much with the content about healthy eating as compared to both family planning and folic acid use. This could be a result of the quality of the adaptation on this topic focused on examples of foods that were mostly unavailable or costly in the local context.

It is possible to reach a large number of young women in Lesotho with digital technologies, as 94% of people aged 18–29 use smartphones. The number of mobile phone connections in 2021 was equivalent to 102.6% of the population (as some people have more than one phone). Seventy-two percent of web traffic is on mobile phones and 95% of web traffic originates from Android Phones. These technologies could serve to expand the reach of health professionals in human resource-restricted settings, making large-scale health promotion efforts possible in countries like Lesotho.^10,11,,^

Barriers to using m-Health technologies for health education include access to public Wi-Fi and data costs. Nthabi was designed in a way that minimized the need for internet access by downloading the entire system including the voice synthesizer that created significant difficulties for downloading. Our data demonstrates that mobile phone use is possible, though, practically, only phones with sufficient available memory could be used. It will become possible for more young women to use Nthabi in the cloud on their phones as Wi-Fi becomes more available.

In our study, participants who were unable to download the intervention to their phones were loaned tablet devices. We purchased 20 devices and loaned them to participants on a rolling basis. At $111 per tablet or $14 per participant this is possibly a cost-effective way to improve health education. Other studies show that cost of m-Health interventions varied substantially based on type and combination of technology used, however, where cost-effectiveness results are reported, the intervention was cost-effective. Future research to determine the cost effectiveness of technologies like Nthabi is required.

The results of our study are limited is several ways. First, the attrition between the number of participants completing the pre- and post-tests could bias the results. While completers and non-completers finished the content of the topic, it is possible that those completing post-tests were more likely to have understood the content. Second, the sample was recruited from only two of the 10 districts of Lesotho and therefore is not a nationally representative sample of women throughout the country. Third, the sample included many participants recruited from the university and vocational schools, and while these participants reported residing in and receiving health services in Berea and Leribe, the results do not reflect women living in rural areas. Further trials are needed to more definitively identify the impact of Nthabi on rural women.

## CONCLUSIONS

The Nthabi conversational agent system increased knowledge of folic acid use and family planning methods among young women in Lesotho. Digital health interventions like Nthabi offer new opportunities to deliver reproductive health information in countries like Lesotho that have limited human resources for health.

## Figures and Tables

**Table 1:** Characteristics of Young Women Enrolled on Nthabi in Lesotho (n=172)

Age (years)	# (%)
18–20	42 (24)
21–23	69 (40)
24–26	44 (26)
27–28	17 (10)
**Marital status**	
Married	15 (9)
Not married	157 (91)
**Level of Education**	
Primary	1 (1)
Secondary	28 (16)
High School	119 (69)
University	24 (14)
**Employment Status**	
Employed	19 (11)
Unemployed	39 (23)
Student	113 (66)
**Recruitment Site**	
Limkokwing University of Technology	34 (20)
Leribe Vocational School	60 (35)
Leribe health facilities	31 (18)
Berea health facilities	7 (4)
*Help Lesotho*	40 (23)

**Table 2.** Participants Viewing a Topic Area, Completing a Topic Area and Taking the Post-Test (n-157)

	# (%) of Participants selecting a topic area	# (%) Participants selecting a topic area who also completed it	# (%) Participants who completed the topic area who also completed the post-test
**Folic Acid**	74 (47.1)	27 (36.5)	27 (100)
**Family Planning**	82 (52.2)	49 (59.8)	26 (53.1)
**Healthy Eating**	76 (48.4)	15 (19.7)	13 (86.7)

**Table 3:** Number of Topic Areas Completed by Participants (n=157)

# of Topics Completed						
**# Participants completing this # of topics**	**Topic Area**	**0**	**1**	**2**	**3**	**Total** ^2^
	Folic Acid	--	7	8	12	27
	Family Planning	--	24	10	15	49
	Healthy Eating	--	1	3	11	15
	Total (#,%)^1^	96 (61.1%)	32 (20.4%)	21 (13.4%)	38 (24.2%)	

1 . # of participants completing the number of topics (in column heading) by topic area, as a % of enrolled participants (n=157)

2 . # of topics completed by the content area

3 . Percentages do not equal 100 due to rounding

**Table 4.** Folic Acid Pre-test and Post-test Responses

	Pretest		Post Test		
	N	# (%) Correct	N	# (%) Correct	p-value
1. What is folic acid?	80	18 (22–5)	31	13 (41.9	0.0502
2. Folic acid is especially important for pregnant women or women who are trying to become pregnant.	80	64 (80.0)	31	31 (100)	--
3. What is the easiest way to get the right amount of folic acid every day?	80	25 (31.3)	31	16 (51.6)	0.0387
4. Folic acid reduces the risk of birth defects.	80	64 (80.0)	31	30 (96.8)	0.0029
5. How much folic acid should women of childbearing age take daily to help prevent birth defects?	79	10 (12.7)	31	21 (67.7)	<.0001
**Total**	399	181 (45.3)	155	111 (71.6)	<.0001

**Response Options** (correct response in **BOLD**)

Q1: (1) Folic acid is a chemical. (2) Folic acid is not a vitamin. (3) Folic acid is a mineral. (4) **Folic acid is a B vitamin.**

Q2: (1) **True**, (2) False

Q3: (1) Drink 2 cups of milk every day, (2) Eat 5 servings of fruits and vegetables, (3) **Take a multivitamin with folic acid**, (4) Drink a lot of water.

Q.4 (1) **True**, (2) False

Q5: (1) 5,000 micrograms (mcg) or 5 milligrams (mg), (2) 1,000 micrograms (mcg) or 1 milligrams (mg), (3) **400 micrograms (mcg) or 0.4 milligrams (mg),** (4) 100 micrograms (mcg) or 0.1 milligrams (mg)

**Table 5.** Family Planning Pre-test and Post-test Responses

	Pre-test		Post-test		
	N	# (%) Correct	N	# (%) Correct	p-value
1. Women can have an operation to avoid having any more children.	90	79 (87.8)	33	32 (97.0)	0.0461
2. Men can have an operation to avoid having any more children.	87	52 (59.8)	33	20 (60.6)	0.9279
3. Women can have a loop or coil placed inside them by a doctor or a nurse.	87	80 (92.0)	33	32 (97.0	0.2377
4. Women can have an injection by a health provider that stops them from becoming pregnant for one or more months.	85	80 (94.1	33	33 (100)	--
5. Women can have one or more small rods placed in their upper arm by a doctor or nurse that can prevent pregnancy for one or more years.	85	75 (88.2)	33	28 (84.8)	0.5979
6. Women can take a pill every day to avoid becoming pregnant.	84	82 (97.6)	33	29 (87.9	0.0999
7. Men can put a rubber sheath on their penis before sexual intercourse.	84	70 (83.3)	32	30 (93.8)	0.0836
8. Women can place a sheath in their vagina before sexual intercourse	84	76 (90.5)	32	31 (96.9	0.0783
9. To avoid pregnancy, women do not have sexual intercourse on the days of the month they think they can get pregnant.	84	56 (66.7)	32	27 (84.4)	0.0367
10. Men can be careful and pull out before climax.	83	77 (92.8)	31	28 (90.3	0.6933
11. As an emergency measure, within five days after they have unprotected sexual intercourse, women can take special pills to prevent pregnancy.	83	69 (83.1)	31	30 (96.8)	0.0106
**Total**	936	796 (85.5)	356	320 (89.9)	0.0233

Response Options: All questions are True or False options; and all correct responses are “true”.

**Table 6.** Healthy Eating Pre-test and Post-test Responses

	Pretest		Post Test		
	N	# (%) Correct	N	# (%) Correct	p-value
What does healthy eating do?	89	39 (43.8)	19	10 (52.6)	0.0455
True or False. You should drink 8 glasses of water or 2 Liters every day.	88	87 (98.9)	19	19 (100)	0.0455
What are the benefits of fiber?	88	67 (76.1)	19	15 (78.9	0.1573
True or False. Calcium is important for having strong bones.	88	84 (95.5)	19	19 (100	0.1573
How many servings of fruits and vegetables should you get each day?	87	29 (33.3)	19	6 (31.6)	0.3173
	**440**	**306 (69.5)**	**95**	**69 (72.6)**	0.4215

**Response Options** (correct response in **BOLD**)

Q1. (1) Helps your body fight illness and diseases, (2) Gives you energy, (3) Helps you focus, (4) **All of the above.**

Q2. (2) **True**, (2) False

Q3. (1) Eating more fiber will improve your skin, (2) Fiber is important for having healthy blood, (3) **Adding more fiber-rich foods to your diet can help you have a healthy digestive system**, (4) Fiber will prevent you from getting sick

Q4. (2) **True**, (2) False

Q5. (1) 2 servings, (2) **5 servings**, (3) 10 servings, (4) Another amount

## Data Availability

The de-identified datasets analysed during the current study are available from the corresponding author on reasonable request.

## Abbreviations

ECA: Embodied Conversational Agent
CONSORT: Consolidated Standards of Reporting Trials
HIV: Human immunodeficiency virus
LMIC: Low Middle Income Country
m-Health: Mobile Health
NTD: Neural Tube Defect
SRH: Sexual and Reproductive Health
STI: Sexually transmitted infections
TB: Tuberculosis
